# Topping influences crop photosynthesis and alters the absorption and redistribution of nutrients: a case study of tobacco

**DOI:** 10.3389/fpls.2025.1597681

**Published:** 2025-07-03

**Authors:** Nuo Shi, Heng Yao, Ge Wang, Gaokun Zhao, Yuping Wu, Guanghai Zhang, Guanghui Kong, Na Wang, Yuxiang Bai, Yu Du, Dingchun Zi, Limei Zhang, Tikun Zhang, Yongping Li, Peng Zhou

**Affiliations:** ^1^ Yunnan Agricultural University, Kunming, China; ^2^ Yunnan Academy of Tobacco Agricultural Sciences, Kunming, China; ^3^ Yunnan Tobacco Company Pu’er City Company, Pu’er, China

**Keywords:** topping, photosynthesis, anatomy, chemical component, redistribution of nutrients

## Abstract

Topping eliminates apical dominance in tobacco, enhancing the quality of the upper leaves. However, the mechanisms by which topping influences photosynthesis and the leaf structure, and regulation of chemical composition remain unclear. This study investigated the regulatory mechanisms of different topping periods (buttoning, budding, first flower, and full flower topping and without topping) on photosynthesis, the leaf structure, and the chemical composition in tobacco by integrating photosynthetic physiology, anatomical, and chemical composition analyses. The results indicated that topping makes the leaves thinner, promotes leaf lamina opening, and results in thicker mesophyll, compact palisade tissue, and loose spongy tissue. It can also significantly enhance photosynthetic capacity. The contents of total sugar, reducing sugar, and K_2_O content increased significantly, while the content of chloride ions decreased significantly. The contents of total nitrogen and nicotine were moderate. In conclusion, topping promotes leaf lamina opening, enhances photosynthesis, and associated with changes in the chemical composition of tobacco leaves. Additionally, this study aims to provide a theoretical basis for the promotion of tobacco topping techniques.

## 1 Introduction

Leaves, as the central organs for plant life activities, are the main site of photosynthesis and directly reflect species’ environmental adaptation strategies through their morphological structures. The structural traits of plant leaves respond dynamically to variations in external environmental conditions, including temperature, moisture, photoperiod, and nutrients (Gao et al., 2023). Tobacco, a heterophyllous plant, has distinct anatomical leaf features, including multiple layers of well-developed palisade cells and highly differentiated spongy tissue, which collaborate to form an efficient photosynthetic system. This specialized palisade and spongy tissue structure enhances the synthesis of carbohydrates and secondary metabolites and provides an ideal interface for the adsorption of flavoring solutions due to its unique intercellular spaces (John et al., 2017). In tobacco, the sink strength of reproductive organs increases during the transition from vegetative to reproductive growth, and mineral nutrients absorbed by the root system and photosynthetic assimilates synthesized by leaves are preferentially transported to sink organs, such as flower buds. This alters the assimilate distribution pattern of the source leaves. During flower bud differentiation, the upper leaves of tobacco plants exhibit significant morphological and anatomical changes. Specifically, decreased cell division activity and inhibited expansion impede leaf morphogenesis. An abnormally high palisade to spongy tissue ratio, which indicates increased leaf compactness, leads to structural deterioration characterized by denser mesophyll cell arrangements and compressed intercellular spaces (Pelivanoska et al., 2014). This imbalance in the source–sink relationship ultimately reduces the rate of biomass accumulation in the upper leaves and disrupts secondary metabolism, decreasing agronomic traits and the sensory quality indicators of tobacco leaves (Aydin and Arslan, 2018). Optimizing the leaf structure and photosynthetic performance through cultivation measures has become a critical scientific challenge for enhancing tobacco leaf quality.

Topping is a common cultivation practice in which the flower heads and young leaves of tobacco plants are removed to eliminate apical dominance, thereby promoting vegetative growth and optimizing resource allocation. Topping alters the growth and material exchange centers of tobacco plants, reconfiguring their original source–sink relationship (Sun et al., 2025). This increases leaf dry matter accumulation and results in more efficient utilization of absorbed nutrients, promoting leaf growth and enhancing tobacco leaf quality. Studies have demonstrated that the upper leaves are most significantly affected by topping (Zhao et al., 2017) and that different topping periods significantly affect the upper leaves. Specifically, buttoning topping results in a dense leaf structure with thicker veins, leading to overly large and thick leaves. In contrast, full flower topping and without topping lead to a looser leaf structure with finer veins, resulting in smaller and thinner leaves. Budding topping and first flower topping promote optimal leaf lamina opening, achieving moderate thickness and better maturity (Xu et al., 2015). The topping period significantly influences the growth, development, and quality of the upper leaves (Li et al., 2016). Topping promotes the accumulation and metabolism of assimilates in tobacco leaves, significantly influencing their chemical composition. In tobacco leaves, early topping (buttoning topping) decreases the reducing sugar content, whereas budding and first flower topping increases the reducing sugar content. Following first flower topping, the reducing sugar content decreases (Fu et al., 2013). Topping promotes root development, enhances root vitality, and increases the nicotine synthesis capacity. Nicotine is transported from the roots to the leaves via the xylem, leading to a higher nicotine content in the leaves (Chen et al., 2019a). Although topping does not increase the final potassium accumulation in tobacco leaves, it significantly reduces potassium inefficient consumption and promotes potassium allocation to the leaves (Liang et al., 2022). Although previous studies have examined the regulation of nitrogen metabolism by topping, the mechanisms underlying the optimization of photosynthetic performance and the extent to which changes in leaf microstructure influence photosynthesis and nutrient distribution remains to be clarified. Therefore, further study is needed to understand the effects of topping at different periods on leaf structural changes, photosynthesis, and the chemical composition of tobacco leaves.

This study used tobacco as the experimental material and employed a combination of plant photosynthetic physiology and anatomy methods to explore the mechanism of the effects of topping on leaf growth, photosynthetic regulation and chemical composition. The results of this study provide a technical foundation for the subsequent promotion of this technology and have significant theoretical and practical implications for promoting the sustainable development of the agricultural industry.

## 2 Materials and methods

### 2.1 Plant materials and treatments

The experimental site is situated in Yuxi, Yunnan Province, China (25°41′19″N, 103°26′13″E; elevation: 1855 m), which has featured a subtropical monsoon climate. The region experiences an average annual temperature ranging from 15.6 to 23.8°C, with annual precipitation ranging from 700 to 900 mm, and average annual sunshine lasting between 2300 and 2700 hours. According to the Chinese classification system for Quaternary Red Clay, the soil is classified as typical red soil, and had the following characteristics: pH 7.32, 1.62% organic matter, 206.74 mg·kg^−1^ alkaline nitrogen, 53.28 mg·kg^−1^ available phosphorus, 235.43 mg·kg^−1^ available potassium.

Tobacco (*Nicotiana tabacum* L., Yunxue No. 38) seeds samples were provided by the Yunnan Academy of Tobacco Agricultural Sciences (Yuxi, Yunnan, China), the seeds were sown in seedling tray that were filled with a mixture of peat and vermiculite (vol/vol, 1:1), carry out floating seedling raising on 8 March 2024. After germination, transplant the seedlings when they have developed their sixth true leaf. The transplanting on 8 May 2024, transplanting plant spacing is 85 cm×35 cm, during planting, 300 kg/hm^2^ of compound fertilizer (N:P_2_O_5_:K_2_O = 12:6:24) was applied. Field management, including irrigation, fertilization, and pest control, followed normal agricultural practices (Ma et al., 2019).

In this experiment, five treatments were set up during the growth process of tobacco from the cone formation period to the full flowering period: buttoning topping (DD1), budding topping (DD2), first-flower topping (DD3), full-flower topping (DD4), and without topping (CK). Each treatment had three blocks with each block covering 0.0067 ha. Fifteen experimental blocks were randomly arranged, totaling 0.1 ha. Each block treatment included at least 100 plants. Sampling was conducted 25 days after each treatment.

Samples were collected between 9:00 and 11:00 on the day of harvest. The 15th leaf from the bottom (out of 20 effective leaves), corresponding to the upper portion of the tobacco plant, was selected for a research object in accordance with the CORESTA guideline (https://www.coresta.org, Based on Agronomy Protocol Section 4.3). Select plants with consistent growth and appearance for measurement. After determining the agronomic traits and photosynthetic characteristics, sampling was conducted. Samples for stomatal characterization and leaf anatomy and ultrastructure observation were preserved in electron microscope fixative solution. Three plant samples from the same plot were mixed to form one biological sample. There were three biological replicates for each treatment. Samples were collected to determine the chemical composition.

### 2.2 Leaf growth, chemical composition, SPAD value, and photosynthetic trait measurements

The length and width of the leaves were investigated, and the length–width ratio was calculated per plant. After freeze-drying the sample, the reducing sugar (RS), total sugar (TS), and starch contents were determined using a Pulse-3000 continuous flow analyzer (Chen et al., 2019b). The total nitrogen (TN) content was determined using the perchloric acid–sulfuric acid digestion method (Dang et al., 2022). The nicotine content was determined by ultraviolet spectrophotometry (5100 PC; Perkin-Elmer) (Chen et al., 2019b). The K_2_O content was determined using flame photometry (Jenway flame photometer model PFP7, Origin UK) (Rana et al., 2019), and the chloride ion (Cl^−^) content was measured using the silver nitrate titration method (Zhang et al., 2021). The cellulose and lignin contents were determined using the FOSS Fibertec 8000^®^ system. The analysis was based on the extraction and weight of neutral detergent fiber, acid detergent fiber, and acid detergent lignin (Dawid and Grzegorz, 2021). The relative chlorophyll concentration (SPAD) was determined in the leaves using a SPAD meter (SPAD-502, Konica-Minolta, Tokyo, Japan). The net photosynthetic rate (Pn), stomatal conductance (Gs), intercellular CO_2_ concentration (Ci), transpiration rate (Tr), and stomatal limitation (Ls) were measured using a portable gas analysis system (LI-COR 6400, LICOR Inc., Lincoln, NE, USA).

### 2.3 Leaf anatomical index measurement

Samples were prepared and fixed for scanning electron microscopy analysis, and stomatal characterization was performed using a modified version of the method described by Zhang et al (Zhang et al., 2011). The samples (1 mm^3^) were cut perpendicular to the main vein, extending from the base of the leaf to one-third of the leaf tip. After gently rinsing with phosphate-buffered saline (PBS), an electron microscope fixative was added. The sample was incubated at room temperature for 2 h and then transferred to 4°C for storage. Subsequently, the samples were fixed in 1% osmium tetroxide prepared with 0.1 M phosphate buffer (pH 7.4) at room temperature for 1 h, away from light. They were then treated sequentially with 30, 50, 70, 80, 95, 100, and 100% ethyl alcohol and isoamyl acetate for 15 min each. The sample was dried in a critical point dryer (K850, QUORUM, UK), placed on conductive carbon film double-sided tape, and sprayed with gold on an ion sputtering sample table (MC1000, HITACHI, Japan) for 30 s. The samples were examined using a scanning electron microscope (SU8100, HITACHI, Japan).

Leaf anatomy measurements were recorded following a modified version of the method described by Ma et al. (Ma et al., 2023). The samples (1 mm^3^) were cut perpendicular to the main vein from the base of the leaf to one-third of the leaf tip. The samples were rehydrated in two changes of BioDewax and Clear Solution, followed by 100% I, 100% II, and 75% alcohol, with each step lasting approximately 5 min. The samples were rinsed with running water and immersed in safranin O staining solution for 15–30 s, followed by rapid dehydration in three cylinders of anhydrous ethanol. The slides were placed in 50, 70, and 80% alcohol for 3–8 s. The samples were then stained in a plant solid green staining solution for 6–20 s and dehydrated in three cylinders of anhydrous ethanol. The samples were transferred to three cylinders of xylene for 5 min. The tissue sections were mounted with neutral balsam. Observations were made under a microscope, and images were captured. The slides were photographed using a NIKON ECLIPSE E100 camera with a 20× magnification lens (NIKON ECLIPSE E100, Nikon, Japan) attached to a NIKON DS-U3 microscope (NIKON DS-U3, Nikon, Japan).

The method described by Sun et al. (Sun et al., 2024). was modified for ultrastructural observation. The samples (1 mm^3^) were sliced perpendicular to the main vein, spanning from the base of the leaf to one-third of its tip. These samples were immediately submerged in a 2.5% glutaraldehyde fixative solution, which was then vacuum-pumped for thorough immersion of the leaf sections. They were then fixed at 0–4°C for 24 h. After fixation, the samples were washed three times with phosphate buffer (pH 7.4), with each wash lasting 15 min. The samples were fixed in 1% osmium tetroxide, prepared using 0.1 M phosphate buffer (pH 7.4) at room temperature, and kept away from light for 7 h. The samples were dehydrated in a series of ethanol concentrations—30, 50, 70, 80, 95, and 100%—with each step lasting 1 h. This was followed by treatment with acetone for 1 h. Ultra-thin sections, with a thickness ranging from 60 to 80 nm, were then cut in a longitudinal orientation using resin blocks and an ultra-thin sectioning machine (Leica UC7, LEICA, Germany). These sections were examined under a transmission electron microscope (either HT7800 or HT7700, HITACHI, Japan). Images were captured for subsequent analysis.

### 2.4 Statistical analysis

The data collected for the length–width ratio, tobacco chemical composition, SPAD value, photosynthetic traits, and leaf anatomy were statistically analyzed using in PASW Statistics 18 (SPSS Inc., Chicago, USA) statistical test with *post hoc* LSD test. After statistical analysis, graphs were generated using Prism v9.0 (GraphPad Prism Software, San Diego, USA). The leaf thickness (LT), upper epidermis thickness (UET), lower epidermis thickness (LET), palisade tissue thickness (PTT), spongy tissue thickness (STT), mesophyll thickness (MT), compactness degree (CD), porosity degree (PD), chloroplast density, quantity and starch density, quantity and size were measured using the acquired images with QuPath v0.4.3 software. A correlation heat map was generated using Metware Cloud (https://cloud.metware.cn).

## 3 Results

### 3.1 Effects of topping on the length–width ratio and tobacco chemical composition

**Figure 1:**
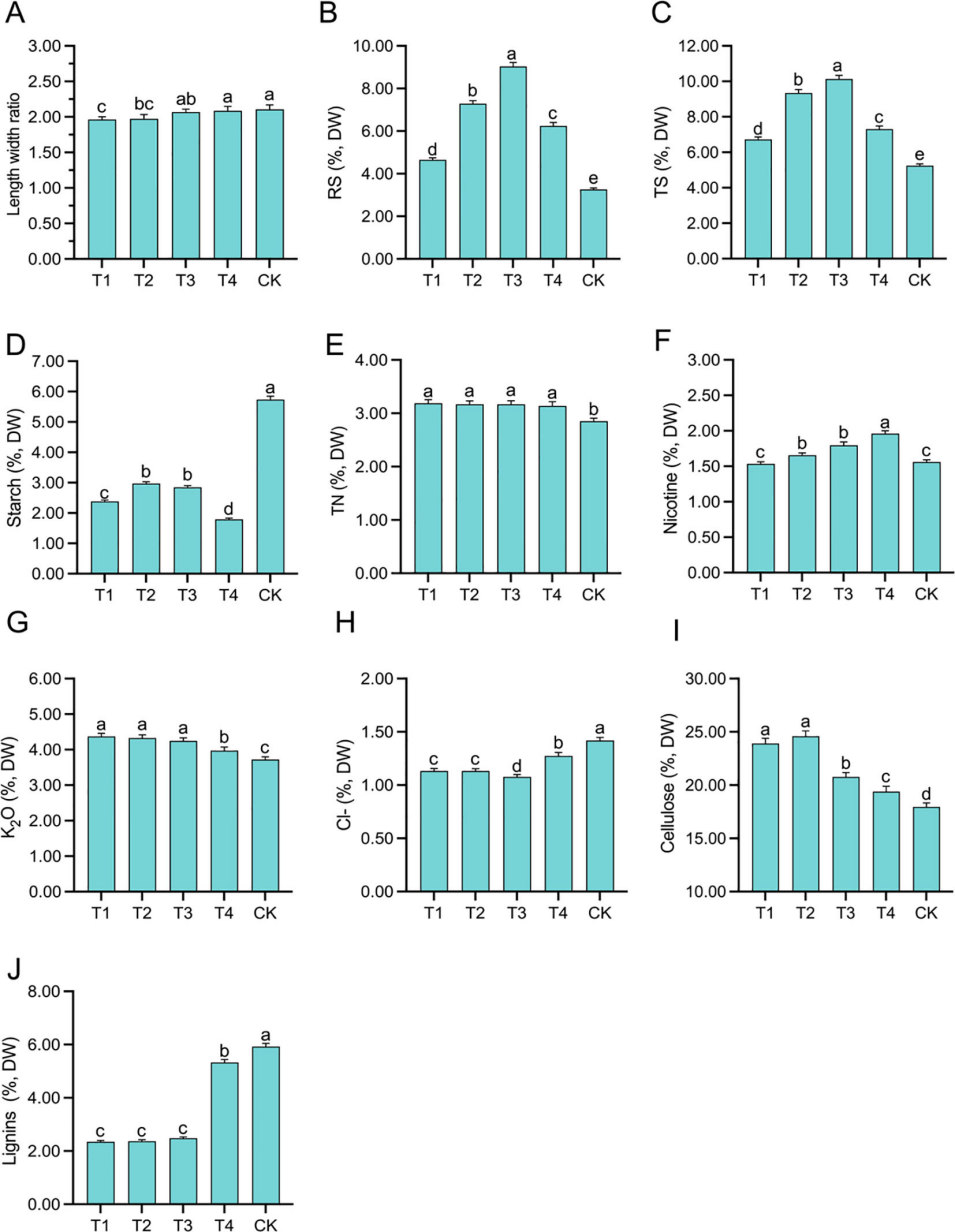
Length–width ratio and tobacco chemical composition affected by topping treatments: length–width ratio **(A)**, reducing sugar (RS) **(B)**, total sugar (TS) **(C)**, starch **(D)**, total nitrogen (TN) **(E)**, nicotine **(F)**, K_2_O **(G)**, Cl^−^ **(H)**, cellulose **(I)**, and lignin **(J)**. The vertical bar indicates the standard deviation of three replications, and different letters indicate significant differences at *p* < 0.05 *post hoc* LSD test. T1–buttoning topping, T2–budding topping, T3–first flower topping, T4–full flower topping, CK–without topping.

The leaf length–width ratio in T4 and CK was relatively large, at 2.09 and 2.11, respectively, while that of T1 and T2 was relatively small, at 1.96 and 1.97, respectively (**Figure 1A**). The RS and TS contents exhibited an initial increase followed by a decrease as the topping period was delayed, with peaks observed at T3 (9.04% for RS and 10.13% for TS). Compared to CK, the RS contents in the four topping treatments increased significantly (*p <* 0.01; *post hoc* LSD test *p <* 0.01) by 42.50% [95% CIs (-1.6331,-1.1411)], 123.21%[95% CIs (-4.2675,-3.7755)], 176.92% [95% CIs (-6.0207,-5.5288)], and 90.82% [95% CIs (-3.2313,-2.7393)], respectively, while the TS contents increased significantly (*p <* 0.01; *post hoc* LSD test *p<* 0.01) by 28.40% [95% CIs (-1.7911,-1.1852)], 78.32% [95% CIs (-4.4077,-3.8017)], 93.35% [95% CIs (-5.1954,-4.5895)], and 38.92% [95% CIs (-2.3670,-1.7610)], respectively (**Figures 1B, C**). The starch content in the topping treatments exhibited an initial increase followed by a decrease, resulting in significant reductions (*p <* 0.01; *post hoc* LSD test *p <* 0.01) of 58.48% [95% CIs (3.2280,3.4812)], 48.19% [95% CIs (2.6378,2.8910)], 50.37% [95% CIs (2.7624,3.0156)], and 68.86% [95% CIs (3.8169,4.0702)] compared to the CK treatment (**Figure 1D**). The TN content in the topping treatments was significantly higher (*p <* 0.01; *post hoc* LSD test *p <* 0.01) than that of CK [95% CIs (-0.1701,0.4076)], and no significant differences were observed among the four topping treatments (**Figure 1E**). The nicotine content was significantly (*p <* 0.05; *post hoc* LSD test *p <* 0.05) the highest in T4, reaching 1.96% [95% CIs (-0.1326,0.4301)], while it was significantly (*p <* 0.05; *post hoc* LSD test *p <* 0.05) the lowest in T1 and CK treatments, at 1.53% [95% CIs (-0.4955,0.0407)] and 1.56% [95% CIs (-0.0017,0.4955)], respectively (**Figure 1F**). The K_2_O content in the topping treatments was significantly (*p <* 0.01; *post hoc* LSD test *p <* 0.05) increased by 17.44% [95% CIs (-0.8072,-0.4909)], 16.18% [95% CIs (-0.7603,-0.4440)], 14.09% [95% CIs (-0.6826,-0.3662)], and 6.35% [95% CIs (-0.4079,-0.0915)] compared to CK (**Figure 1G**). The Cl^-^ content in the topping treatments was significantly (*p <* 0.01; *post hoc* LSD test *p <* 0.05) reduced by 20.13% [95% CIs (-0.2388,0.3325)], 20.22% [95% CIs (0.2401,0.3338)], 24.03% [95% CIs (0.2941,0.3878)], and 10.50% [95% CIs (0.0979,0.1916)] compared to CK (**Figure 1H**). The cellulose content in the topping treatments was significantly (*p <* 0.01; *post hoc* LSD test *p <* 0.05) increased by 33.09% [95% CIs (-6.7630,-5.1282)], 36.94% [95% CIs (-7.4546,-5.8198)], 15.61% [95% CIs (-3.6228,-1.9881)], and 7.64% [95% CIs (-2.2532,-.6184)] compared to CK (**Figure 1I**). The lignin content in T4 and CK was relatively high, at 5.33% [95% CIs (-3.7044,0.1643)] and 5.92% [95% CIs (-3.5901,0.2787)], respectively (**Figure 1J**).

### 3.2 Effects of topping treatment on SPAD values and photosynthetic traits

**Figure 2:**
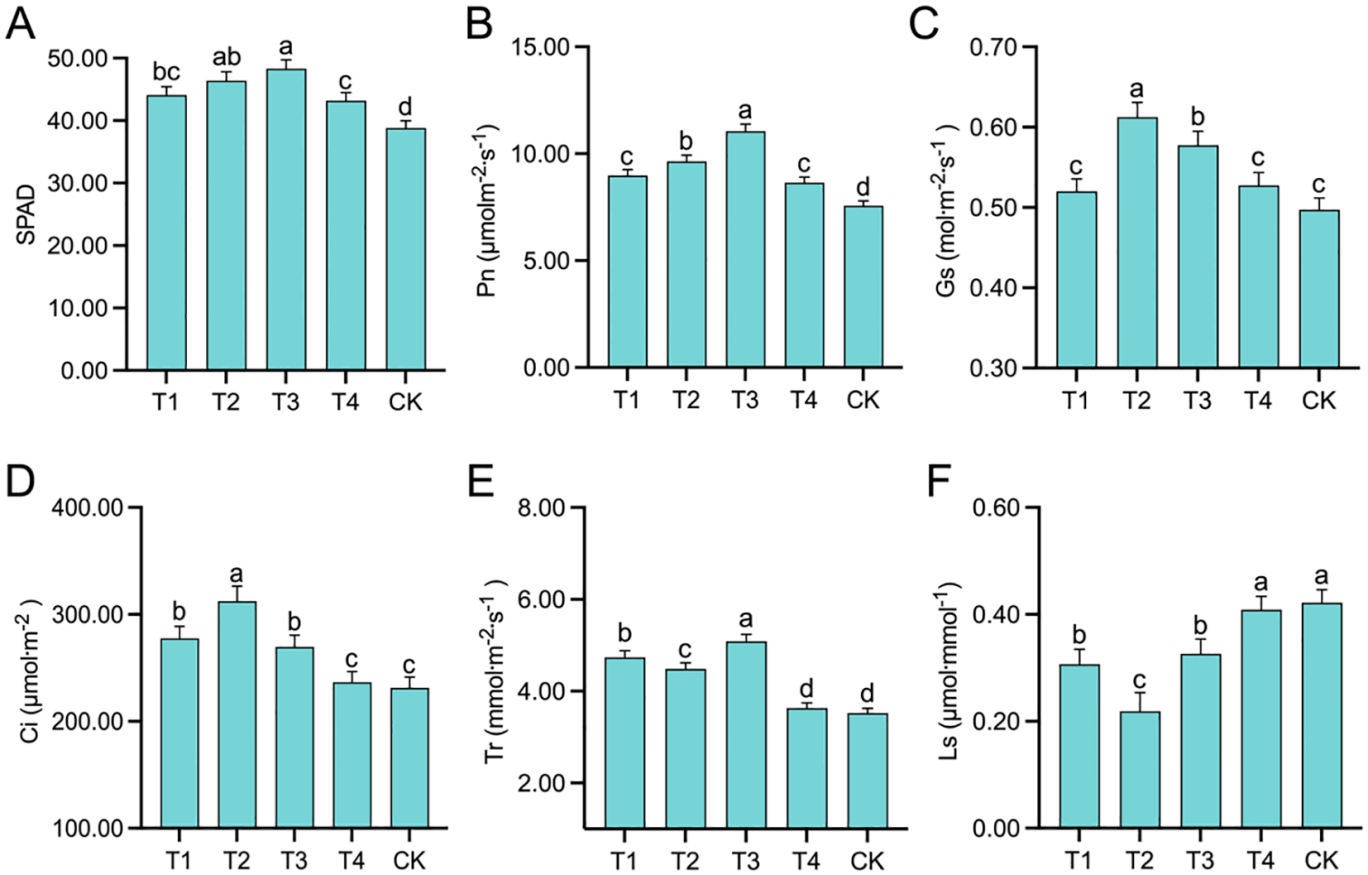
Photosynthetic traits affected by different topping treatments: The relative chlorophyll concentration (SPAD) **(A)**, net photosynthetic rate (Pn) **(B)**, stomatal conductance (Gs) **(C)**, intercellular CO_2_ concentration (Ci) **(D)**, transpiration rate (Tr) **(E)**, and stomatal limitation (Ls) **(F)**. The vertical bar indicates the standard deviation of three replications, and different letters indicate significant differences at *p* < 0.05 *post hoc* LSD test. T1–buttoning topping, T2–budding topping, T3–first flower topping, T4–full flower topping, CK–without topping.

The SPAD values of the leaves (**Figure 2A**) exhibited an initial increase followed by a decrease. The topping treatments significantly (*p <* 0.01; *post hoc* LSD test *p <* 0.05) increased the SPAD values by 13.66% [95% CIs (-7.7166,-2.8834)], 19.59% [95% CIs (-10.0166,-5.1834)], 24.48% [95% CIs (-11.9166,-7.0834)], and 11.34% [95% CIs (-6.8166,-1.9834)] compared to the CK treatment. As the topping period was delayed, Pn (**Figure 2B**), Gs (**Figure 2C**), and Ci (**Figure 2D**) exhibited an initial increase followed by a decrease. Compared to CK, the topping treatments significantly (*p* < 0.05; *post hoc* LSD test p < 0.01) increased Pn by 18.67% [95% CIs (-1.9182,-0.9084)], 27.47% [95% CIs (-2.5838,-1.5740)], 46.00% [95% CIs (-3.9863,-2.9765)], and 14.18% [95% CIs (-1.5781,-0.5683)]; Gs by 4.63% [95% CIs (-0.0529,0.0069)], 23.22% [95% CIs (-0.1453,-0.0855)], 16.22% [95% CIs (-0.1105,-0.0507)], and 6.10% [95% CIs (-0.0602,-0.0004)]; and Ci by 19.87% [95% CIs (-0.0602,-0.0004)], 35.04% [95% CIs (-66.6253,-25.3225)], 16.48% [95% CIs (-101.7336,-60.4308)], and 2.21% [95% CIs (-58.7841,-17.4813)]. Tr (**Figure 2E**) was significantly (*p* < 0.01; *post hoc* LSD test p < 0.01) higher under T1, T2, and T3 treatments than under T4 and CK treatments, and Tr was relatively similar under T4 and CK treatments. Ls was lowest under the T2 [95% CIs (-0.2543,-0.0361)] treatment (**Figure 2F**) and that of the T4 [95% CIs (0.0309,0.2415)] and CK [95% CIs (0.0437,0.2543)] treatments was relatively high and similar. These results indicate that as the topping period was delayed, the SPAD values and photosynthetic parameters, including Pn, Gs, Ci, and Tr, exhibited an initial increase followed by a decrease. Notably, these values under T2 and T3 treatments were relatively higher.

### 3.3 Effects of topping treatment on leaf anatomy, stomatal characterization, and ultrastructural observation

**Figure 3:**
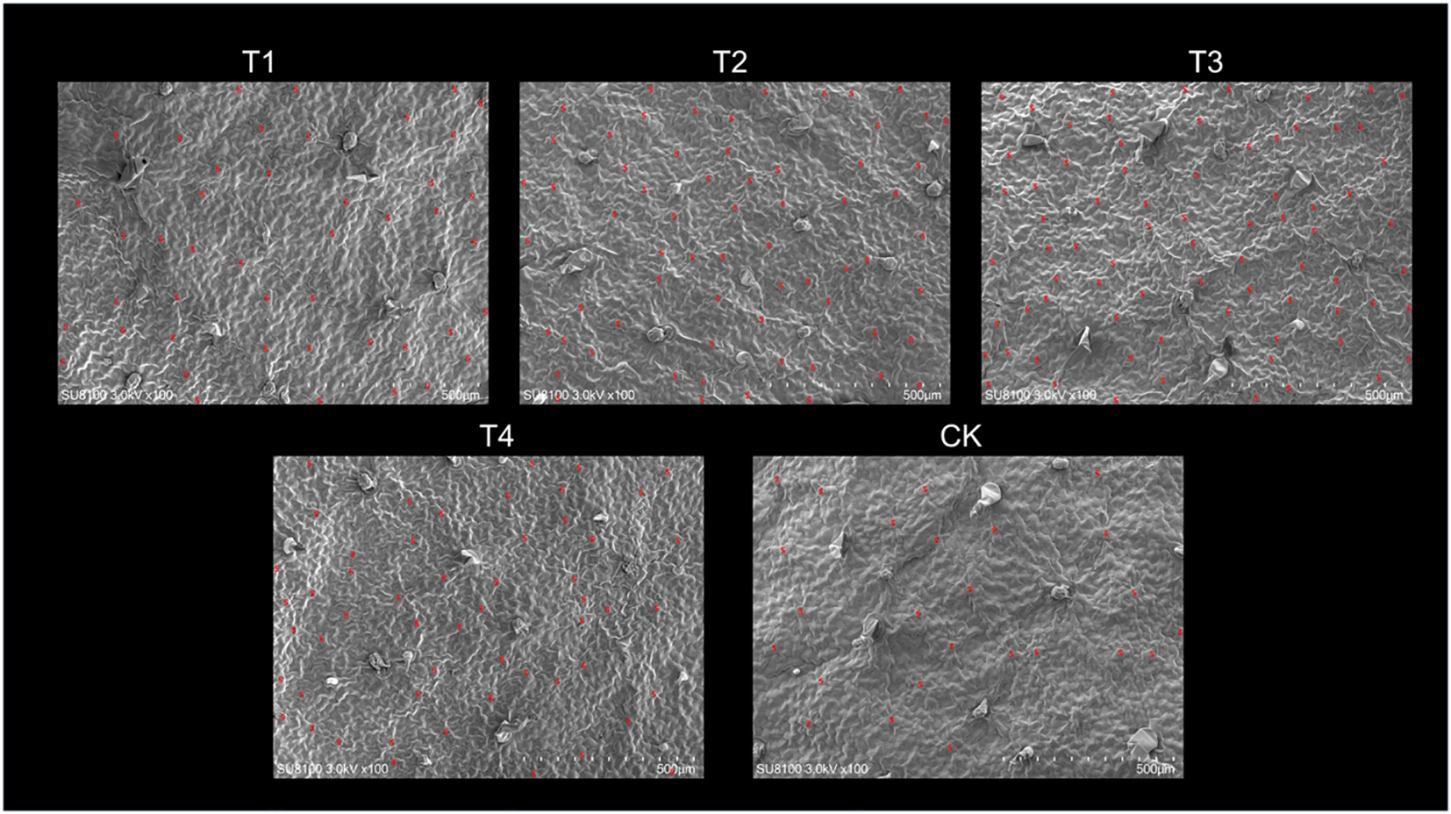
Leaf stomata in response to different topping treatments. The five treatments are T1 (buttoning topping), T2 (budding topping), T3 (first flower topping), T4 (full flower topping), and CK (without topping). Visual field area: 1,184,836.38 µm^2^; scale bar: 50 µm.

**Figure 4:**
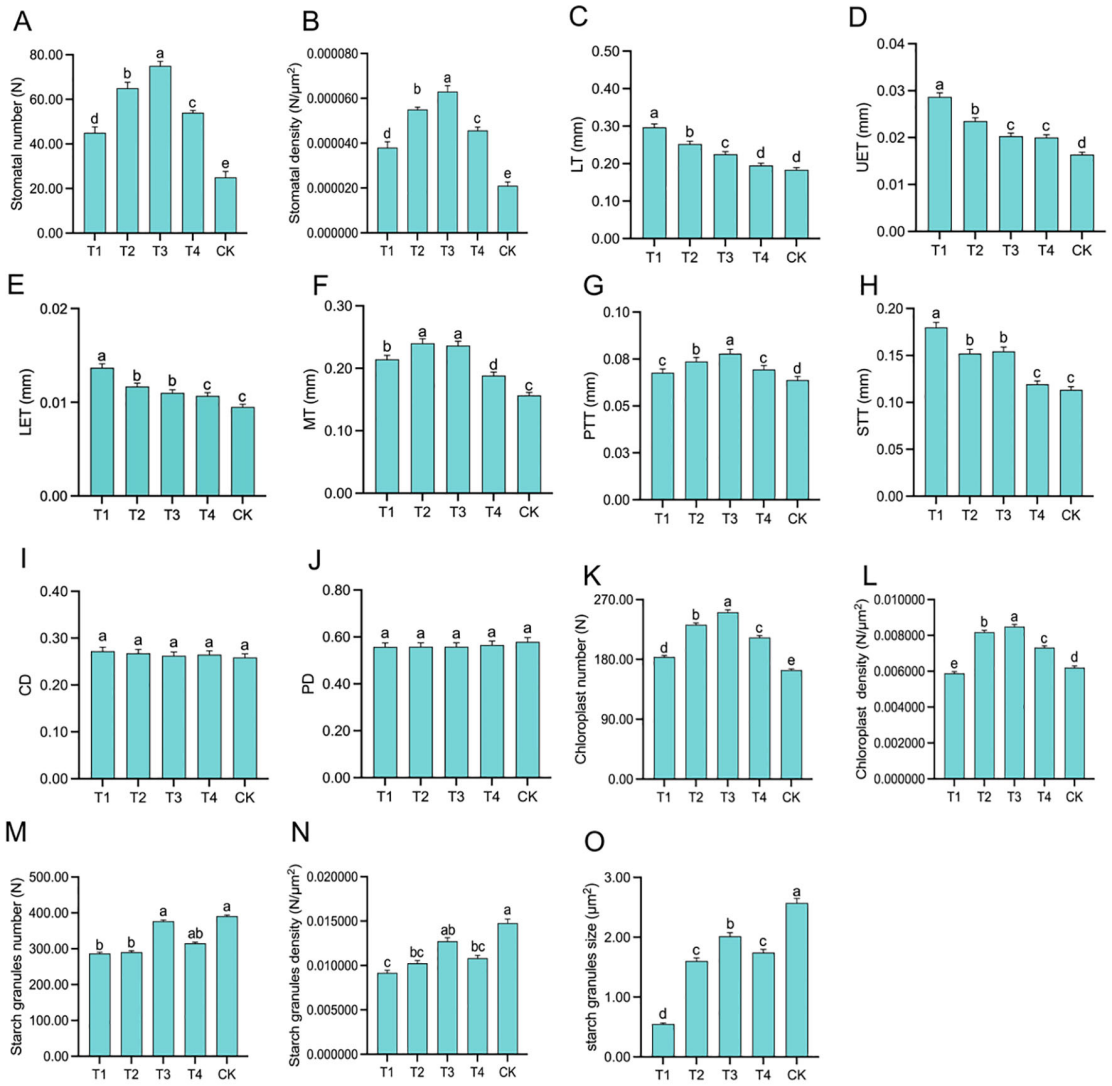
Leaf structure in response to different topping treatments: stomata numbers **(A)**, stomata density **(B)**, leaf thickness (LT) **(C)**, upper epidermis thickness (UET) **(D)**, lower epidermis thickness (LET) **(E)**, mesophyll thickness (MT) **(F)**, palisade tissue thickness (PTT) **(G)**, spongy tissue thickness (STT) **(H)**, compactness degree (CD) **(I)**, and porosity degree (PD) **(J)**, chloroplasts numbers **(K)**, chloroplasts density **(L)** starch granules numbers **(M)**, starch granules density **(N)**, starch granules size **(O)**. The vertical bar indicates the standard deviation of three replications, and different letters indicate significant differences at *p* < 0.05 *post hoc* LSD test. T1–buttoning topping, T2–budding topping, T3–first flower topping, T4–full flower topping, CK–without topping.

Scanning electron microscope (SEM) images of the leaves revealed that the number and density of stomata were closely associated with topping treatments (**Figure 3**). As the topping period was delayed, the number and density of stomata exhibited an initial increase followed by a decrease, with both parameters peaking at T3, reaching a stomatal number of 75 (**Figure 4A**). Compared to CK, the topping treatments significantly (*p <* 0.01; *post hoc* LSD test *p <* 0.01) increased stomatal density by 80.95% [95% CIs (-0.00002067,-0.00001333)], 161.90% [95% CIs (-0.00003767,-0.00003033)], 200.00% [95% CIs (-0.00004567,-0.00003833)], and 119.05% [95% CIs (-0.00002834,-0.00002100)] (**Figure 4B**).

**Figure 5:**
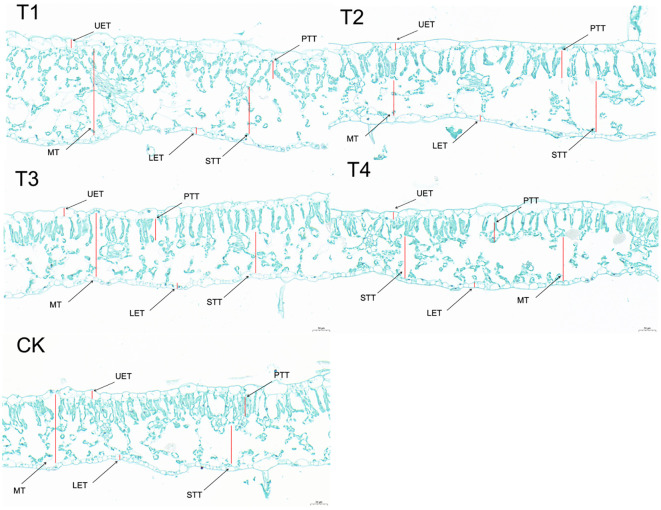
Leaf structure images under different topping treatments. The five treatments are T1 (buttoning topping), T2 (budding topping), T3 (first flower topping), T4 (full flower topping), and CK (without topping). LE, leaf thickness; UET, upper epidermis thickness; LET, lower epidermis thickness; PTT, palisade tissue thickness; STT, spongy tissue thickness; MT, mesophyll thickness; CD, compactness degree; PD, porosity degree. Scale bar: 50 µm.

Leaf anatomy cross-sections (**Figure 5**) revealed that as the topping period was delayed, LT (**Figure 4C**) gradually decreased. UET (**Figure 4D**) and LET (**Figure 4E**) exhibited a gradually decreasing trend. MT (**Figure 4F**) initially increased and then decreased, with T2 and T3 showing relatively higher values of 0.2402 mm and 0.2364 mm, respectively. PTT (**Figure 4G**) initially increased and then decreased under the topping treatments. However, compared to CK, the PTT were significantly (*p <* 0.01; *post hoc* LSD test *p <* 0.05) higher by 6.09% [95% CIs (-0.00774188,-0.00003145)], 15.49% [95% CIs (-0.01373522, -0.00602478)], 22.02% [95% CIs (-0.01790188,-0.01019145)], and 8.75% [95% CIs (-0.00943855,-0.00172812)] for T1, T2, T3, and T4, respectively. The STT (**Figure 4H**) of the topping-treated plants were consistently higher than those of the CK, measuring 0.1798 mm, 0.1520 mm, 0.1544 mm, and 0.1194 mm for T1, T2, T3, and T4, respectively. As the topping period was delayed, the compactness of the palisade tissue and the uniformity of the spongy tissue (ST) initially increased and then decreased. Additionally, the cell gaps gradually decreased (**Figure 5**). In addition, leaf anatomy cross-sections revealed the degree of tightness or looseness in the leaf structure. As the topping period was delayed, CD (**Figure 4I**) gradually decreased, while PD (**Figure 4J**) gradually increased.

**Figure 6:**
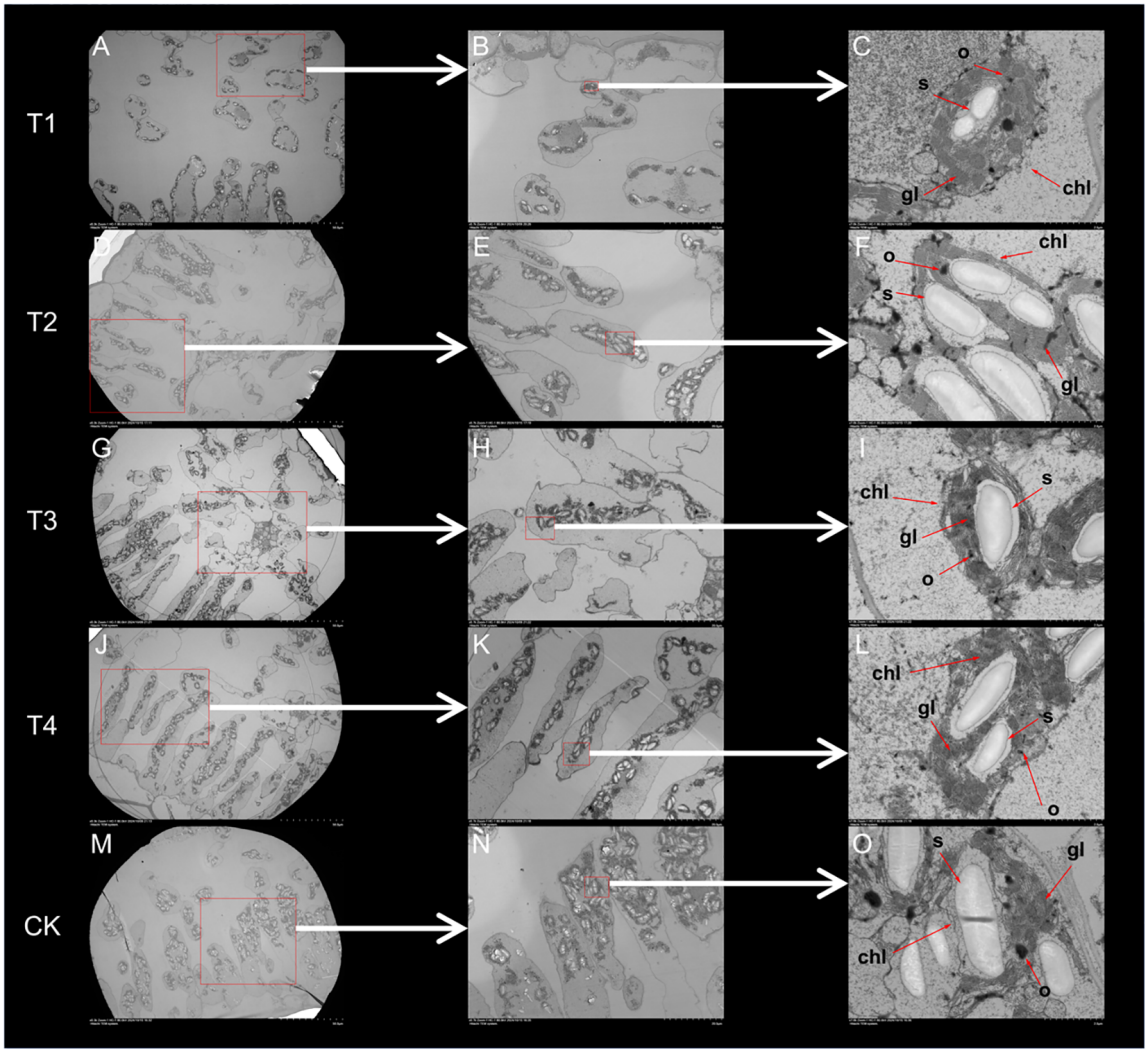
Leaf ultrastructure affected by different topping treatments. T1: **(A–C)**, T2: **(D–F)**, T3: **(G–I)**, T4: **(J–L)**, CK: **(M–O)**. chl–chloroplast, gl–grana layer, o–osmiophilic particles, s–starch grains. T1–buttoning topping, T2–budding topping, T3–first flower topping, T4–full flower topping, CK–without topping.

Based on the leaf ultrastructure (**Figure 6**), the distribution of organelles across treatments was uniform, while their number gradually increased as the topping period was delayed. The differences in the number and distribution of chloroplasts were particularly evident (**Figures 6A, B, D, E, G, H, J, K, M, N**). In treatment T1 (**Figure 4K, L**), chloroplasts were widely distributed but present in smaller numbers and density. Treatments T2 and T3 (**Figure 4K, L**) exhibited a wide distribution and higher chloroplast numbers and density. In contrast, chloroplasts in treatments T4 and CK (**Figure 4K, L**) were more concentrated, exhibited higher chloroplast numbers and density. Compared to CK, the topping treatments significantly (*p <* 0.01; *post hoc* LSD test *p <* 0.05) increased chloroplast numbers by 12.20% [95% CIs (-24.5029,-14.8305)], 41.46% [95% CIs (-72.8362,-63.1638)], 53.05% [95% CIs (-91.8362,-82.1638)], and 29.88% [95% CIs (-53.8362,-44.1638)].

The internal chloroplast structure also exhibited significant changes (**Figure 6C, F, I, L, O**). Under topping treatment, the gaps between the chloroplast grana lamellae were reduced, and vacuoles were present within the chloroplasts. However, as the topping period was delayed, the chloroplast basal lamellae became increasingly condensed, resulting in a more dense and compact structure, with the eventual disappearance of vacuoles. This phenomenon, known as chloroplast basal lamellae densification, was particularly pronounced in the T4 treatment, which may reflect structural changes associated with chloroplast development. In the chloroplasts of CK treatment, the chloroplast basal lamellae appeared blurred and dissolved, while the chloroplast grana lamellae exhibited swelling, disintegration, and disordered arrangement, accompanied by membrane structure damage. These changes led to starch granule leakage, which became freely dispersed in the cytoplasm outside the chloroplasts.

Chloroplasts in all treatments contained starch granules and osmiophilic particles, but the number of starch granules varied significantly among treatments (**Figure 6C, F, I, L, O**). Compared to CK, the topping treatments significantly (*p <* 0.01; *post hoc* LSD test *p <* 0.01) increased starch granules number by 26.60% [95% CIs (30.6155,178.7178)], 25.58% [95% CIs (26.2822,174.3845)], 3.58% [95% CIs (-60.0511,88.0511)], and 19.44% [95% CIs (2.2822,150.3845)] (**Figure 4M**). Under the topping treatments, the size of starch granules initially increased and then decreased. The starch granules in the CK treatment were enlarged, occupying the thylakoid distribution area. Compared to CK, the topping treatments significantly (*p <* 0.05; *post hoc* LSD test *p <* 0.05) increased starch granules size by 78.60% [95% CIs (1.05355522,2.99068411)], 37.64% [95% CIs (-0.00016345,1.93696545)], 21.62% [95% CIs (-0.41230045,1.52482845)], and 32.26% [95% CIs (-0.13859578,1.79853311)] (**Figure 4O**). These results indicate that as the topping period was delayed, the stomatal number and density initially increased and then decreased and leaf anatomy became progressively more compact. Chloroplast densification gradually intensified under the topping treatments, while the chloroplast structure exhibited signs of damage.

### 3.4 Correlation analysis between the photosynthetic index and leaf anatomy

**Figure 7:**
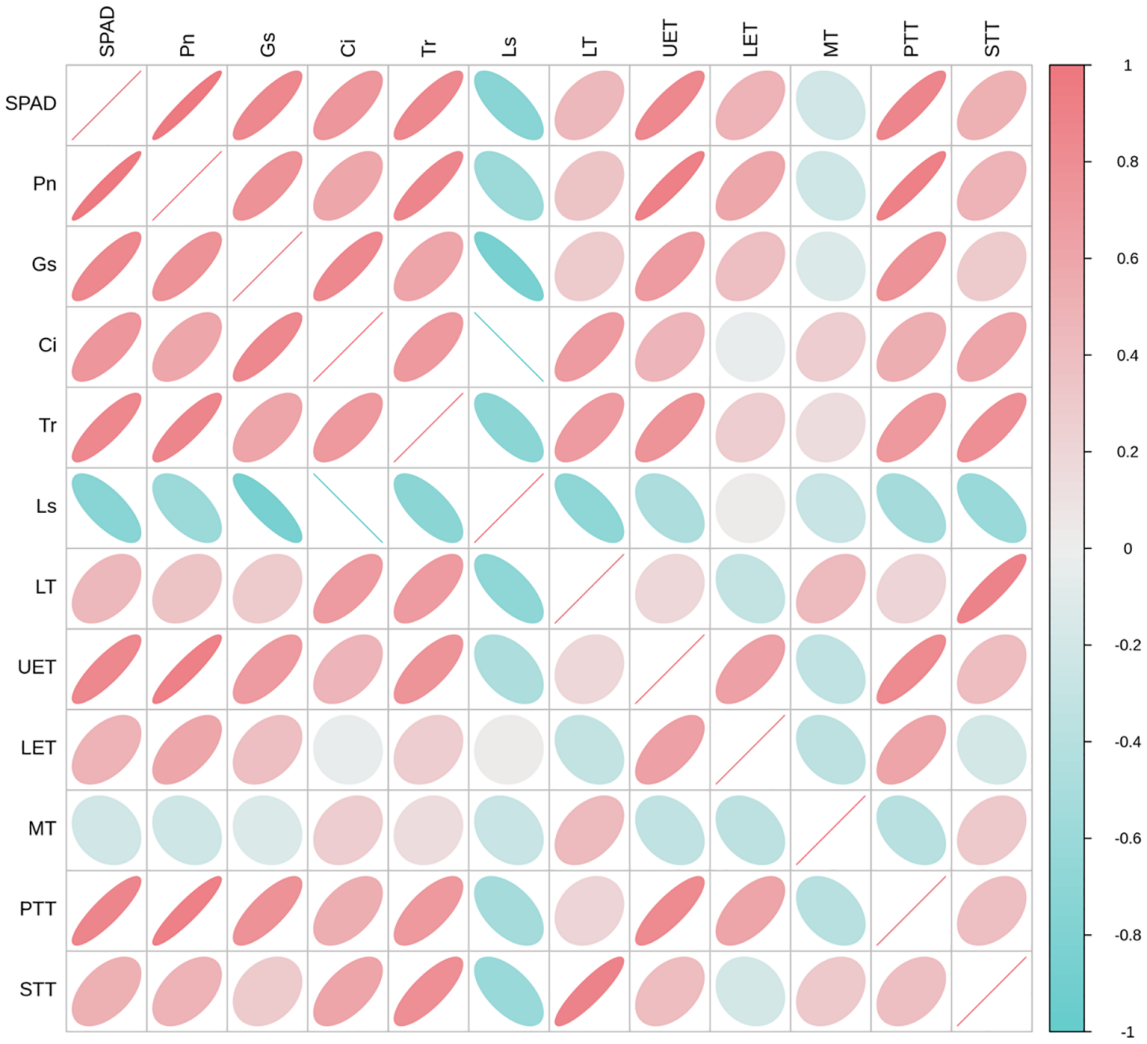
Correlation analysis between the photosynthetic index and leaf anatomy traits. The relative chlorophyll concentration–SPAD, net photosynthetic rate–Pn, stomatal conductance–Gs, intercellular CO_2_ concentration–Ci, transpiration rate–Tr, stomatal limitation–Ls, LE, leaf thickness; UET, upper epidermis thickness; LET, lower epidermis thickness; MT, mesophyll thickness; PTT, palisade tissue thickness; STT, spongy tissue thickness.

Correlation analysis was conducted between the photosynthetic index and leaf anatomy traits (**Figure 7**). SPAD values, Pn, and Gs were positively correlated with all leaf anatomical indices, except MT, with correlation coefficients (R) ranging from 0.285 to 0.918. SPAD, Pn, and Gs were significantly correlated with UET and PPT. Ci was negatively correlated with LET and positively correlated with all other leaf anatomical indices, with R values of 0.464–0.693. Tr was positively correlated with all leaf anatomical indices, with R values ranging from 0.148 to 0.795. Ls was positively correlated with LET and negatively correlated with all other leaf anatomical indices, with R values ranging from −0.272 to −0.693. These results demonstrate a positive correlation of SPAD values and photosynthetic parameters (Pn, Gs, Ci, and Tr) with leaf anatomical indices (LT, UET, PTT, and STT).

## 4 Discussion

This study found that the ratio of leaf length to width was larger in the topping treatment than in the without topping treatment, indicating that topping is beneficial for leaf lamina opening. As an important structure controlling water transpiration and carbon dioxide absorption, the density, quantity and size of stomata affect the rate of gas exchange and transpiration (Jiang et al., 2011; Meng and Fricke, 2017). This study shows that leaves under topping treatment have a greater number and higher density of stomata, as well as larger Gs and smaller LS. This leads to an increase in the amount of carbon dioxide entering the leaves (Ci increase), providing more substrates for the dark reaction, enhancing the carbon dioxide assimilation rate, and increasing Pn. While photosynthesis is enhanced, Tr also increases, regulating the transpiration of the leaves. Although these indicators suggest an improvement in photosynthetic performance, we need to further verify this through measurements such as the A/Ci curve or chlorophyll fluorescence.

The structure of leaf tissue is closely related to the intensity of photosynthesis. This study found that compared with non-topping, topping would make the mesophyll of leaves thicker, the palisade tissue more compact, and the spongy tissue looser. Leaves with thick mesophyll facilitate the rapid transfer of metabolites among mesophyll cells, which is conducive to enhancing photosynthetic capacity. The slender and closely arranged palisade cells can enhance light capture ability through light transmission and multi-directional scattering. Meanwhile, the loose spongy tissue and its intercellular spaces help to scatter and reflect light, increasing the probability of light absorption and improving the utilization rate of light energy (Nobel and Hartsock, 1981; Li et al., 2020; Muhammad et al., 2020; Ren et al., 2019; John et al., 2017). As topping time was delayed (T1~T4), the thickness of mesophyll and the compactness of palisade tissue first increased and then decreased, while the looseness of spongy tissue first decreased and then increased. Among them, the leaf structure was better when topping was appropriately delayed (T2 and T3), and Pn first increased and then decreased (T1~T4). Therefore, topping changed the leaf tissue structure and enhanced photosynthesis.

This study found that the SPAD value increased with topping treatment. As the topping period was delayed (T1~T4), the chloroplast basal lamella became increasingly compact, the gaps in the chloroplast grana lamella gradually decreased, and the chloroplast basal lamella in the leaves gradually became looser and more uniform, with the thylakoid stacks neatly arranged. A greater number of well-shaped chloroplasts can enhance light absorption, providing a favorable site for enzyme distribution and metabolite exchange (Raju et al., 2024), promoting the transfer and transformation of chloroplast functions, increasing the photosynthetic reaction rate of leaves, and facilitating the formation and accumulation of photosynthetic products (Fitzpatrick et al., 2022; Sun et al., 2022). In the leaves without topping treatment, the chloroplast basal lamella was blurred and dissolved, and the chloroplast grana lamella was swollen, disintegrated and disordered in arrangement, the observed chloroplast disorganization may be associated with reduced efficiency of light energy capture and transfer by chloroplasts and weakened photosynthesis (Liu et al., 2021). This study also found that the number and size of starch granules in chloroplasts of leaves with topping treatment were less, while the opposite was true for leaves without topping treatment. Excessive starch granules would compress and damage the photosynthetic membrane and also block the light source, thereby hindering the absorption of light by the photosynthetic membrane and causing a decline in photosynthetic intensity (Vandromme et al., 2023).

The formation of chemical components is the result of the combined effects of variety, ecological conditions, and cultivation measures. Top-dressing is an important cultivation measure that affects chemical components (Zou et al., 2024). Top-dressing alters the intensity of photosynthesis in leaves, influencing the synthesis of photosynthetic products. Among these, sugar substances and cell wall components in photosynthetic products are significant parts of the chemical composition of tobacco leaves (Gui et al., 2024). In treatments T1, T2 and T3, topping was carried out when the leaves were in a vigorous nutritional period. As the topping time was delayed, Pn continuously increased, and the accumulated nutrients rose, resulting in an upward trend in the contents of TS, RS and starch. However, in treatment T4, the tobacco plants were in the full-flower stage, Pn weakened, the apical dominance was significant, and the reproductive growth was vigorous, leading to the concentration of nutrients in the reproductive organs and a decrease in the nutrient content in the leaves, with the contents of TS, RS and starch declining. The content of TS and RS and Pn in CK treatment were lower than those in topping treatment, while the starch content was much higher than that in topping treatment. These changes in sugar content may reflect altered source–sink relationships and nutrient allocation dynamics following topping (Smith and Stitt, 2010). Excessively low sugar content in tobacco leaves would make the smoke rough and more irritating, while excessively high sugar content would make the smoke tasteless. Low starch content in fresh leaves affects the transformation of carbohydrate substances during the curing process, which may lead to insufficient sugar content in tobacco leaves during combustion, resulting in a lack of rich taste and flavor. However, high starch content may mean more potential sugar sources, but excessive starch content can cause too much smoke and tar to be produced during smoking of the cured tobacco leaves, affecting the taste and quality (Chen et al., 2021). The cellulose content of tobacco leaves is relatively high and the lignin content is relatively low after topping. The opposite situation occurs when topping is not performed. The microfibril network formed by cellulose provides a framework support for tobacco leaves, enhances the smoldering holding time of cigarettes, and the porous structure can absorb environmental moisture to reduce the drying and fragmentation of tobacco strands, improving the integrity during the processing. During the aging process, cellulose is degraded by microorganisms to generate low-molecular sugars, which participate in the Maillard reaction to produce caramel aroma. However, the pyrolysis of lignin generates phenolic substances such as phenol, increasing the roughness of smoke and reducing the smoldering property (Yamaguchi et al., 2019). Topping increases the contents of RS, TS and cellulose in tobacco leaves, reduces the contents of starch and lignin, which is beneficial to the subsequent tobacco leaf curing and processing, and improves the quality of cigarettes.

Carbohydrates are the main products of photosynthesis, while nitrogen, potassium and chloride ions are inorganic nutrients absorbed by plants from the soil through their root systems. Topping, by eliminating apical dominance, significantly affects the processes of nutrient absorption, utilization and redistribution in plants (Aydin and Arslan, 2018). Compared with without topping, topping treatment results in a higher nicotine content. This was because after topping, the demand for nutrients by the apical sink of the tobacco plant is eliminated, and the root system of the plant, which was originally a source, becomes a sink, promoting secondary growth of the root system (Fu et al., 2013). As a result, a large amount of nicotine synthesized by the roots is transported to the leaves. In topping treatment, the nicotine content was T1 < T2 < T3 < T4. This was because early topping led to the tobacco plants absorbing nitrogen from the soil and converting it into nutrients for plant growth, thereby reducing the effective accumulation of nicotine in the leaves (Lei et al., 2022). Nicotine is the most important secondary metabolite in tobacco, playing a crucial role in stimulating the nervous system and inducing pleasure in smokers (Ma et al., 2019). However, an excessively high nicotine content can increase the irritancy of tobacco leaves and is not conducive to safety (Yang et al., 2022).

In addition, the experimental results revealed that the total nitrogen content increased after topping. This was attributed to the fact that topping activated the activity of enzymes related to nitrogen metabolism, promoting nitrogen assimilation and utilization, which led to an increase in TN (Kurt and Kinay, 2021). Topping increases the K_2_O content. Topping eliminates the apical dominance, and the nutrients (including K) originally supplied to the top will be redistributed to the leaves, thereby increasing the K_2_O content (Hasanuzzaman et al., 2018). Relevant studies have shown that an increase in potassium leads to an increase in Gs, thereby increasing Tr (Sperling et al., 2024). In this study, the K_2_O, Gs, and Tr in the topping treatment were all higher than those in the without topping treatment. The observed increases in potassium content and stomatal conductance suggest that topping may enhance K_2_O content and water use efficiency. Potassium can increase the elasticity and flexibility of tobacco leaves, enhance their burning power and holding power, and reduce the tar content (Hu et al., 2021). Topping also reduces the Cl^-^ content in tobacco leaves. Excessive accumulation of Cl^-^ can damage the cell structure of leaves, causing the collapse of the intercellular spaces in the spongy tissue, which hinders gas diffusion and combustion oxidation reactions, resulting in the deterioration of the burning properties of tobacco products and a reduction in the smoldering time (Wang et al., 2020). Topping can increase the contents of nicotine, TN, and K_2_O in tobacco leaves, while reducing the content of Cl^-^.

## 5 Conclusions

This study, with a focus on photosynthetic physiology and anatomical analysis, systematically discussed the mechanism of the effects of topping on leaf growth, photosynthetic regulation and chemical composition. We found that topping made the leaves thinner, increased the degree of leaf lamina opening, thickened the mesophyll, made the palisade tissue tighter, loosened the spongy tissue, increased the number and improved the morphology of chloroplasts, enhanced photosynthesis and improved the chemical composition of tobacco leaves. Based on anatomical characteristics combined with photosynthetic physiological regulation, this study proposed the mechanism of the impact of topping on the photosynthesis of tobacco and the redistribution of nutrient absorption. In future research, we will clarify the molecular mechanism by which topping affects tobacco through multi-omics analysis, and the topping was analyzed to examine its impact on the tobacco root system, which will provide valuable references for tobacco cultivation.

## Data Availability

The original contributions presented in the study are included in the article/supplementary material. Further inquiries can be directed to the corresponding authors.
